# Effects of the “Inspirational Lecture” in Combination With “Ordinary Antenatal Parental Classes” as Professional Support for Expectant Parents: A Pilot Study as a Randomized Controlled Trial

**DOI:** 10.3389/fpubh.2020.00285

**Published:** 2020-07-28

**Authors:** Stina Thorstensson, Anette Ekström-Bergström, Caroline Bäckström

**Affiliations:** ^1^School of Health Sciences, University of Skövde, Skövde, Sweden; ^2^Department of Health Sciences, University West, Trollhättan, Sweden

**Keywords:** transition, pregnancy, childbirth experience, parenthood, sense of coherence, couple relationship, QDR36

## Abstract

**Background:** Both expectant mothers and their partners describe weaknesses in ordinary parental preparatory professional support provided internationally and nationally within Sweden. Therefore, it is necessary to develop the parental preparatory professional support provided by midwives for expectant parents within Sweden. This study will evaluate the effects on expectant parents of receiving a combination of an “inspirational lecture” and “ordinary antenatal parental classes” compared with only “ordinary antenatal parental classes.”

**Methods/Design:** This block randomized controlled trial included an intervention as a pilot study, in which expectant parents were randomized for (1) the inspirational lecture *and* ordinary antenatal parental classes (intervention group [IG]) (*n* = 66) or (2) ordinary antenatal parental classes (control group [CG]) (*n* = 60). Data collection with repeated questionnaires was conducted in the first week and 6 months after birth. Statistical analyses were conducted for participant characteristics, differences between parents within IG and CG, effects of the intervention, intention to treat, and internal consistency of the included measurements.

**Results:** The intervention showed a tendency to be gainful for one out of four outcomes related to birth experience, and parents' perceived quality of parental couple relationship consensus and sexuality and manageability. These results were more prominent for the partners. Parents within both the intervention and control groups reported decreased social support in the first 6 months after birth.

**Conclusion and Clinical Implications:** Overall, the concept of the inspirational lecture in combination with ordinary antenatal parental classes as parental preparatory professional support seems to be a valuable care intervention. However, this study was a pilot study and the results should therefore be interpreted with caution. More research is needed since childbirth and transition to parenthood are complex processes in need of comprehension.

## Introduction

Becoming parents for the first time is a major change of life event (1), a transition that involves the physical endeavors of pregnancy, birth, and breastfeeding (2) but also changes in social roles and roles for the parental couple (1, 3, 4). Parents-to-be can be unaware of the challenges posed by pregnancy, childbirth, and parenthood (5). Negative experiences from childbirth are, for example, associated with deterioration of maternal health (6), development of postpartum depression (7), and problematic bonding between mother and infant (8). The challenges of transitioning to parenthood may result in decreased quality within the parental couple's relationship (3, 9–12), with increased risk of separation (13). Positive experiences from birth are, on the other hand, associated with women feeling empowered and encouraged in their motherhood (14). Such positive experiences have previously been described as related to the mothers' personal strength and sense of control and coherence (15). Individuals' ability to manage their own health and to cope with everyday problems plays a key role in sense of coherence (SOC) (16), which also seems to be affected by parents' transition to parenthood (9, 17, 18).

To handle the challenge of transition to parenthood, both first-time mothers and partners need parental preparatory support, particularly socially and professionally (19–21). However, partners to first-time mothers describe that professional support in counseling during pregnancy mainly focuses on the woman and the physiological changes, and partners felt left out and ignored (22). Also, professional support in antenatal parental classes is mainly focused on physiological changes, while parents want more focus on parenthood and partner relationships (23). Internationally, antenatal parental classes are given various names, such as expectant parent classes, antenatal parenthood education, antenatal education, childbirth classes, and antenatal classes. For this study, the term “ordinary antenatal parental classes” is used.

To prepare for parenthood, first-time mothers describe the importance of different kinds of professional support (19). Professionals should support parents' couple relationship during the transition to parenthood (24), and such support could have a positive effect on communication between the couple (19, 20). Professionals, such as midwives, who provide professional support for parents need to have knowledge about parents' supportive needs. They also need competence to be attentive to parents' individual needs (25, 26), since the parents may not be able to express their needs themselves. Both professional and social support has been described as interactive processes that affect well-being and health (27, 28). However, social support is offered within the individual's social network and needs to be based on working relationships and trust. Professional support, on the other hand, is directly available but limited by professional domain and knowledge. Professional support could be considered as a care intervention and should focus on strengthening the individual's access to social support (27).

Internationally, professional support offered during pregnancy varies, depending on the country. For example, some countries offer antenatal parental classes, while others do not. International studies have also found that professional support during pregnancy leads to increased knowledge and better preparation for labor and birth (29) and improved infant care (30). In Sweden, first-time mothers and partners are offered professional support together during antenatal care and in-group sessions (ordinary antenatal parental classes), mostly provided by midwives (31). Research shows weaknesses in the professional preparatory support offered to parents; therefore, it is important to increase knowledge about such support. Since autumn 2012, midwives who work in a hospital labor ward in central Sweden have provided an inspirational lecture as a large-group parental class for expectant parents. The purpose of the lecture is to give the parents a more satisfactory preparation for birth and safer experience during birth as well. Previous research on parents' perceptions of the lecture is that their ability to absorb adequate information is increased by the pedagogically mediated information provided through role-play by the midwives (19, 20). Also, the humor used by the midwives who provide the lecture makes the parents laugh at something they, in fact, are nervous about (giving birth to a child). The laughter helps the parents to relax and understand the information provided. Besides this, parents perceive that the lecture facilitates their understanding of how to prepare for birth and parenthood together with their partner. This understanding facilitates the parental couple's ability to communicate with each other, which contribute to their feelings of togetherness (19, 20). However, there is no previous research on the effect of the inspirational lecture on parents. Further research is therefore needed to gain deeper knowledge about the effects of professional support for expectant parents.

The present study tested the inspirational lecture as an intervention to be provided in combination with ordinary antenatal parental classes as parental preparatory support. The aim of the present study was to evaluate the effects of expectant parents receiving a combination of the inspirational lecture and ordinary antenatal parental classes compared with expectant parents receiving only ordinary antenatal parental classes. The study was guided by hypotheses that the inspirational lecture in combination with ordinary antenatal parental classes would have an effect on first-time mothers' and partners' (1) birth experience (primary outcome), (2) breastfeeding and skin-to-skin contact, (3) perceived professional support, (4) perceived quality of the parental couple's relationship, (5) perceived social support, (6) sense of coherence, and (7) parent-to-infant relations and feelings (secondary outcomes).

## Methods

### Trial Design and Participants

This is a randomized control trial that was performed as a pilot study with a follow-up design (32). Participants were randomly assigned in blocks (i.e., through block randomization) to one of two groups and received professional support through (1) the inspirational lecture provided by midwives as a large-group parental class in combination with ordinary antenatal parental classes provided by midwives (intervention group) or (2) ordinary antenatal parental classes provided by midwives (control group).

Participants gave their consent before randomization. To select trial participants, the following inclusion criteria were used: (1) first-time mother with her partner, (2) singleton pregnancy between gestational weeks 24 and 35, (3) intention to give birth at the county hospital, and (4) ability to understand and speak the Swedish language. The sampling plan was predisposed by a time aspect, which was to recruit participants between May 1 and June 1, 2015. Therefore, a consecutive sampling was performed to recruit all of the parents who met the inclusion criteria over the specific time interval (32). Based on calculations, we intended to include 200 expectant parents (expectant first-time mothers *n* = 100; partners *n* = 100) within this study. In total, we targeted 100 parents for the intervention group and another 100 parents for the control group, which corresponds to an allocation ratio of 1:1. Furthermore, we targeted an equal randomization regarding gender since the two parents within the same parental couple were randomized to the same group (intervention or control group).

### Settings

This study was conducted in a county in southwestern Sweden, consisting of urban, suburban, and rural districts. The county hospital labor-ward sees an average of around 3,500 births per year. Within this county, as well as nationally in Sweden, pregnant women are offered professional support free of charge through the Swedish public primary healthcare system (31). Within the setting for the present study, professional support is offered within midwifery care in terms of prenatal assessments at antenatal units *and* ordinary antenatal parental classes.

### Stratification, Randomization, and Participant Recruitment

**Figure 1:**
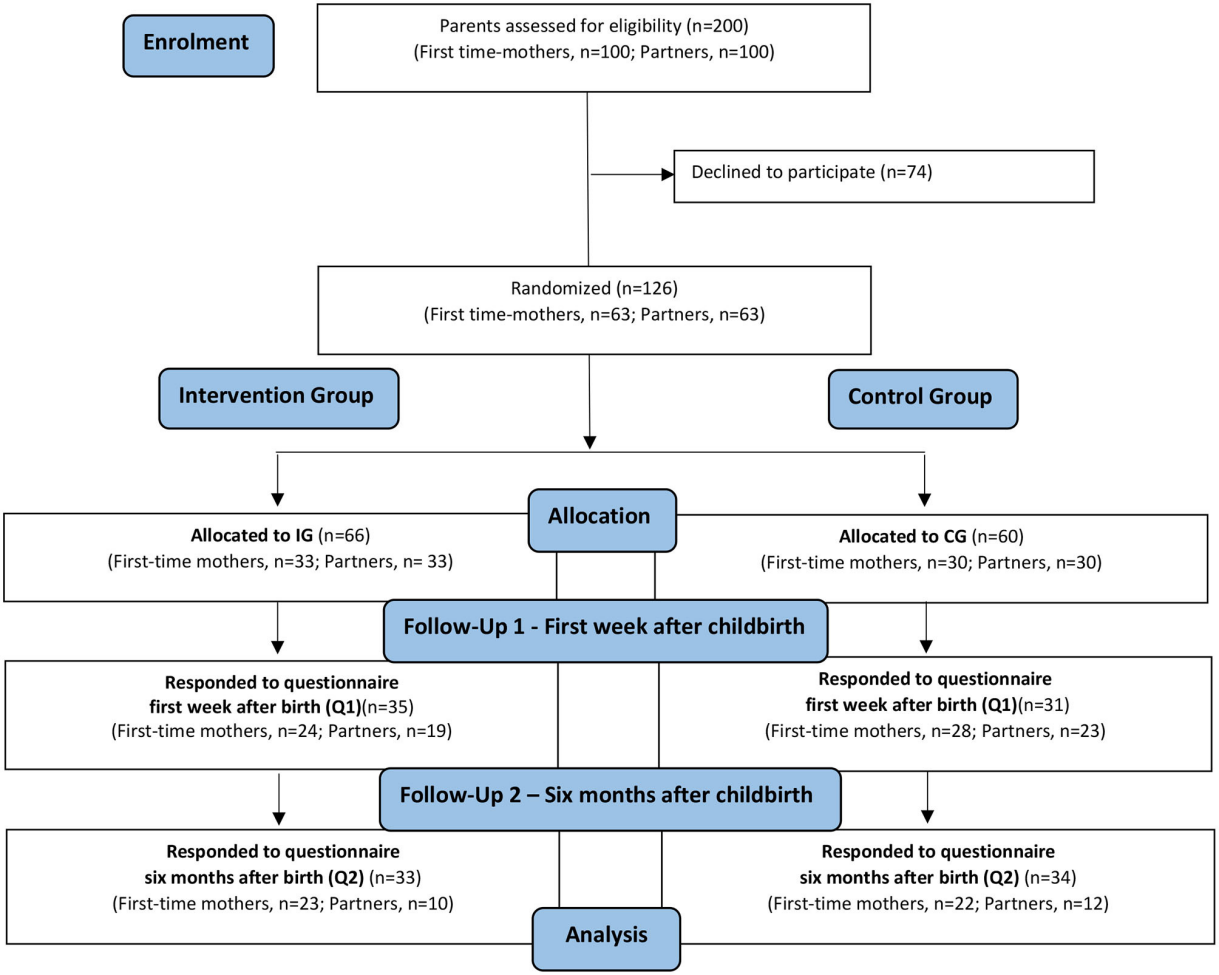
Flow diagram of participation allocation, follow-up, and data analysis in the randomized controlled trial.

Eligible parents who met the inclusion criteria were provided information about the study from midwives who worked at the three antenatal units. The information was provided both orally and in writing to the parents during a routine control (prenatal assessment) with the midwife. Among those parents who agreed to participate in the study, block randomization was performed to allocate the participants into groups that resulted in equal sample sizes. We used an intention-to-treat approach since we aimed to keep participants who were randomized in the groups to which they were assigned. To achieve this, a box was presented that included sequentially numbered sealed envelopes with either (1) a ticket for both the inspirational lecture and a ticket for ordinary antenatal parental classes (IG) or (2) a ticket to ordinary antenatal parental classes (CG). The midwives who handled the sealed envelopes did not know which envelopes contained a ticket to the inspirational lecture and which envelopes did not. To participate in the inspirational lecture, a ticket was needed, which made it possible to control that only those parents included in the IG received the intervention. The randomization was carried out directly after the parents had agreed to participate. The midwives were instructed beforehand to provide the parents with envelopes in numbered order, starting with the lowest number. This was to ensure that the randomization was correctly performed without impact by the midwives. The midwives noted the number of the specific envelope on the specific parent's completed consent form. This was to control for whether the parent was randomized to an IG or CG. A flow diagram of how parents were allocated to the IG and CG is shown in Figure 1.

### Intervention

The intervention consisted of the inspirational lecture as a parental class for expectant parents provided as a complement to the ordinary antenatal parental classes. The ***intervention group (IG)*** consisted of parents who received a combination of the following: (1) the inspirational lecture as a large-group parental class provided by midwives and (2) ordinary antenatal parental classes provided by midwives at antenatal units. The CG consisted of parents who received only ordinary antenatal parental classes provided by midwives at antenatal units. The intervention will be explained in detail below.

#### The Inspirational Lecture

For the intervention, the hospital introduced the inspirational lecture as a large-group parental class for expectant parents. The lecture was originally developed and provided at another hospital that was not included in this study. At the time of this study, the inspirational lecture was not provided elsewhere within Sweden (except at the hospital that developed the lecture). In total, four midwives were trained as providers of the inspirational lecture at the hospital. Those four midwives were taught to provide the lecture in pairs, which gave two pairs in total. The inspirational lecture is a professional support provided by midwives for expectant parents as a large-group lecture. During the inspirational lecture, midwives who work within antenatal and/or labor care explain how parents can prepare for birth. The information is focused on normal birth and how parents can strengthen their individual as well as mutual skills as a parental couple to be able to handle the challenges that come with childbirth (labor). For example, midwives strive to present childbirth (labor) as a normal life event that the parents themselves can prepare to cope with. The midwives use a pedagogical approach that includes role-playing to increase the parents' understanding. By role-playing, the midwives who provide the inspirational lecture illustrate the pregnant woman's and the partner's perspective. This is to make both perspectives visible and to emphasize the value of each as well. The inspirational lecture lasts for 2 h. Within the present study, the inspirational lecture was provided on two specific dates for the parents who were randomized for the IG. Only expectant parents who were in the IG received the inspirational lecture.

#### Ordinary Antenatal Parental Classes

According to routines already established within the setting, ordinary antenatal parental classes were offered to expectant parents. These classes were given in accordance with the national Swedish guidelines (31). For the intervention within the present study, these classes were included, without any changes from how they already were provided for expectant parents. Midwives at antenatal units provided these parental classes four to five times during pregnancy, in groups of six to eight parental couples and one midwife. During these classes, parents were provided with information about pregnancy, labor, breastfeeding, parenthood, and relationships between parents.

### Measurements and Data Collection

The primary and secondary outcomes were measured at (1) first week (Q1) and (2) 6 months after birth (Q2). Q1 was provided to the parents by the midwives at the postnatal ward of the hospital. The parents answered Q1 in paper format. The parents filled out Q2 using the web-based computer system titled Education Survey Automation Suite (EvaSys). Q2 was sent to the parents via email 6 months after the birth. For those participants who did not answer Q1 or Q2, one reminder was sent at each of the time points. The primary outcomes were the parents' birth experience measured by questions selected from the short version of the intrapartal-specific QPP questionnaire (QPP-I) (33) at Q1 and Q2. To assess parents' perceived birth experience, four questions (items) were included: “*I had a positive birth experience*”; “*I had a normal birth*”; “*I perceived that I had control during birth*”; and “*I perceived myself being safe during birth*.” Each item was a four-point response scale ranging from 1 (“I do not agree at all”) to 4 (“I completely agree”). Two questions that concerned the subjective importance of the parents' birth experience were also included from the QPP questionnaire: “*This is how important it was to me to have a positive birth experience*” and “*This is how important it was to me to have a normal birth*.” Each item was a four-point response scale ranging from 1 (“of little or no importance”) to 4 (“of the very highest importance”). In total, six items were included within Q1 and Q2. All the items included also a “not applicable” response alternative. Each item was calculated separately within this study; the higher the score, the more positive the experience (Table 1).

**Table 1:** Measurements and time of measurements.

**Measurements**	**First week after childbirth (Q1)**	**Six months after childbirth (Q2)**
Socio-demographic characteristics	X	
Birth experience	X	X
Breastfeeding	X	X
Skin-to-skin contact	X	
Mother Perceived Professionals Support scale (MoPPS scale)	X	
Sense of Coherence (SOC-13)	X	X
Quality of Dyadic Relationship (QDR36)	X	X
The Multidimensional Scale of Perceived Social Support (MSPSS)	X	X
Mother to Infant Relations and Feelings scale (MIRF-scale): *First section:* Parent's perceived relation to the child *Second section:* Parent's perceived feelings for the child	X	X

*Socio-demographic characteristics: age; gender; employment; perceived economy*.

*Birth experience: 6 items calculated separately. Each item score range 1–4, the higher the score, the more positive birth experience*.

*Breastfeeding: 3 items concerning whether or not the mothers were breastfeeding, as well as exclusive and partly breastfeeding*.

*Skin-to-skin contact: 1 item concerning the parent's skin-to-skin contact with the child during the child's first period of alertness after birth (first 2 h after birth)*.

*Mother Perceived Professionals Support scale (MoPPS scale): 8 items. Index is the sum of all items. Index score range 8–56, the higher the score, the stronger the perceived professional support*.

*Sense of Coherence (SOC-13): 13 items, divided into three dimensions: Comprehensibility (5 items); Manageability (4 items); Meaningfulness (4 items). Index is the sum of all items. Index score range 13–91, the higher the score, the higher Sense of Coherence*.

*Quality of Dyadic Relationship (QDR36): 36 items, divided into five dimensions (score range 1–6): Dyadic Consensus (11 items); Dyadic Cohesion (4 items); Dyadic Satisfaction (11 items); Dyadic Sensuality (5 items); Dyadic Sexuality (5 items). Index is the sum of mean values from all dimensions. Index score range 5–30, the higher the score, the higher perceived quality of dyadic relationship*.

*The Multidimensional Scale of Perceived Social Support (MSPSS): 12 items, divided into three dimensions (score range 1–7): Family (4 items); Friends (4 items); Significant others (4 item). Index is the sum of all items. Index score range 12–84, the higher the score, the higher perceived social support*.

*Mother to Infant Relations and Feelings scale (MIRF-scale): First section (Parent's perceived relation to the child): 7 items. For first-time mothers index is the sum of all items, index score range 7–49. For partners index is the sum of 6 items (question about breastfeeding excluded from index), index score range 6–42. The higher the score, the stronger perceived relation to the child. Second section (Parent's perceived feelings for the child): 7 items. Index is the sum of all items. Index score range 7–49, the higher the score, the stronger perceived parent's feelings for the child*.

The secondary outcomes were data of obstetric and neonatal outcomes collected from electronic medical records and psychometric scales measuring psychosocial variables. Data for birth (obstetric and neonatal) outcomes were collected through the first-time mothers' hospital medical records. Data of obstetric outcome included duration of labor (hours between onset of contractions/labor start and birth); dilation of the cervix (centimeters) when arriving at labor ward; type of birth (spontaneous vaginal birth, vacuum extraction, forceps, or cesarean section); and gestational week at birth. Data on neonatal outcomes consisted of the Apgar score. Questions concerning breastfeeding were included within the questionnaires. The first-time mothers responded to questions whether they were breastfeeding (1) *during the child's first period of alertness (first two hours after birth)*; (2) *at one week and six months after birth*; and (3) *exclusively* (child was given only breastmilk) *or partly* (child was given both breastmilk and formula). Included within Q1 was also a question about skin-to-skin contact between the parent and child during the child's first period of alertness (first 2 h after birth): “*Did you have skin-to-skin contact with your child during his/hers first period of alertness after birth?*” Response alternatives were “yes,” “no,” *or* “I do not remember” (Table 1).

All psychometric instruments included within the questionnaires were available in Swedish, and most of them have been validated previously with good internal consistency. The *MoPPS scale* (Mother Perceived Professionals Support scale) is a seven-graded Likert scale, ranging from 1 to 7, developed to assess mothers' perceived experiences with professional support (34). The scale is validated through interviews with parents (22, 35–38). The scale consists of one question about how professional support from healthcare professionals, such as midwives, is perceived. This question is followed by eight statements, such as “*sensitive/not at all sensitive*,” “*understanding/not at all understanding*,” and “*were calmed/were stressed*.” The index was a calculated summary of the total score of the eight items. The higher the score, the more positive the perceived experience with professional support. Within the current study, the MoPPS scale was used to measure parents' perceived experiences with professional support from both (1) healthcare professionals at the labor ward and (2) healthcare professionals at the postnatal ward (Table 1).

To assess the parents' perceived quality of parental couple relationship, the validated Quality of Dyadic Relationship (QDR36) scale was used (3, 39). QDR36 consists of 36 items scored with Likert scales from 1 to 6. The scale covers five dimensions: *Dyadic Consensus, Dyadic Cohesion, Dyadic Satisfaction, Dyadic Sensuality*, and *Dyadic Sexuality*. The index score is the total sum of the mean values from the separate dimensions; the higher the score index, the stronger the person's estimated perceived quality of couple's relationship (Table 1).

To assess parents' perceived social support, the Swedish version (40) of the Multidimensional Scale of Perceived Social Support (MSPSS) (41, 42) was used. The scale used within this study was previously validated among women with hirsutism and nursing students (40). In total, the scale consists of 12 items (seven-point Likert scales ranging from 1 to 7) that cover three dimensions: *family, friends*, and *significant others*. The index is calculated by summarizing the total score for all items; the higher the score, the stronger the perceived social support (Table 1).

To assess the parents' sense of coherence, the validated Swedish version of the SOC-13 was used (43). The SOC-13 consists of 13 items scored with Likert scales ranging from 1 to 7 (16, 44). The scale covers three dimensions: *comprehensibility, manageability*, and *meaningfulness*. The index score is the total sum of all items; the higher the score, the higher the person's estimated sense of coherence (Table 1).

The seven-graded Likert MIRF scale (Mother to Infant Relations and Feelings scale) (ranging from 1 to 7) was used to assess the parents' relation toward and feelings for their child (34, 45–47). The scale is validated through interviews with parents (34). The MIRF scale consists of two different sections that assess (1) the parent's perceived relation to his/her child and (2) the parent's perceived feelings for his/her child. Within the first section, the parent's perceived relationship to the child is assessed through seven different statements, such as: “*I talk a lot with my baby/I do not talk at all with my baby*” and “*I enjoy resting when my baby is with me/I enjoy resting when my baby is with someone else*” and so on. The index is calculated by summarizing the seven items; the higher the score, the stronger the perceived relation to the child between the parents. The first section of the MIRF scale includes a question about breastfeeding: “*I enjoy breastfeeding/I do not enjoy breastfeeding*.” For the first-time mothers, this question is included within the index score, plus it is calculated and analyzed separately. For the partners, the question regarding breastfeeding was not included in the calculation of the score index for the partner's perceived relation to his/her child. For analysis, the variable that describes the first section of the MIRF scale is named “*Parent's perceived relation to the child*.” Within the second section of the MIRF scale, the parent's feelings for his/her child are assessed with a question concerning the parent's perceived contact with the child. Seven different items (seven-graded response rate, ranging 1 to 7) constructed of opposing word pairs follow the question: “*warm/cold*,” “*secure/insecure*,” “*close/distant*,” “*confident/unconfident*,” “*stable/unstable*,” “*easy/difficult*,” and “*pleasant/unpleasant*.” The index is calculated by summarizing the seven items. The higher the grade, the stronger the parent's perceived feelings for the child. The variable that describes the index score of the second part of the MIRF-scale is named “*Parent's perceived feelings for the child*” within the current study (Table 1).

Socio-demographic characteristics were self-reported within Q1 (Table 1). Before the current study, two pilot studies were conducted to test parents' experiences form responding to questionnaires in paper and web-based form. The results of the pilot studies showed that the information included in the questionnaires and the composition of the same was generally understandable and manageable.

### Data Analysis

Statistical analyses were conducted using the Statistical Package for the Social Sciences (SPSS) version 22 (IBM, Corp, Armonk, NY, USA). Descriptive statistics were conducted to describe the socio-demographic characteristics of the participants. The index and dimensions for the measurements were presented as a mean (M) and dispersion by standard deviation (SD) (Table 2). To analyze the questionnaires and the effects of the intervention on first-time mothers and partners, the Mann–Whitney U-test was performed. To analyze differences between parents within IG and CG, the Mann–Whitney test was used for ordinal variables, and the chi-squared test was performed for discrete variables. Cohen's guidelines were used to interpret clinical change when a significant result was achieved from Mann–Whitney; the effect was defined as small (η2 > 0.01), medium (η2 > 0.06), or large (η2 > 0.14) (48). Analyses were carried out for first-time mothers and partners separately, as well as mutually as a group of parents within the IG or CG. Intention-to-treat analysis was performed for comparison of changes over time within groups using the non-parametric Friedman's test for the continuous variables: birth experience, QDR36, MSPSS, and SOC-13. The Wilcoxon signed-rank test for *post hoc* testing was performed after a statistically significant Friedman's test. To evaluate the internal consistency for the index of the scales, *MoPPS, QDR36, MSPSS, SOC-13, parent's perceived relation to the child*, and *parent's perceived feelings for the child*, Cronbach's alpha was calculated. *P* ≤ 0.05 were considered significant, and *p* ≤ 0.1 were interpreted as tendencies.

**Table 2:** Overview of characteristics for first-time mothers and partners within the intervention (IG) and control group (CG) at different times (Q) throughout the study.

	**IG**		**CG**	
	**First-time mothers** **(*n* = 24)**	**Partners** **(*n* = 19)**	**First-time mothers** **(*n* = 28)**	**Partners** **(*n* = 23)**
	***n* (%)**	***n* (%)**	***n* (%)**	***n* (%)**
**Age**				
≤25 years	9 (37.5)	1 (5.3)	2 (7.1)	1 (4.3)
26–34	12 (50.0)	6 (31.6)	15 (53.6)	4 (17.4)
≥35	2 (8.3)	2 (10.5)	4 (14.3)	7 (30.5)
Missing	1 (4.2)	10 (52.6)	7 (25.0)	11 (47.8)
**Marital status**				
Cohabiting	16 (66.7)	16 (84.2)	16 (57.1)	15 (65.2)
Not cohabiting	2 (8.3)	1 (5.3)	0 (0)	0 (0)
Missing	6 (25.0)	2 (10.5)	12 (42.9)	8 (34.8)
**Perceived economy, at Q1**				
Very good	3 (12.5)	4 (21.1)	0 (0)	1 (4.3)
Good	10 (41.7)	9 (47.4)	14 (50.0)	11 (47.8)
Sufficient	5 (20.8)	4 (21.1)	2 (7.1)	3 (13.0)
Strained	0 (0)	0 (0)	0 (0)	0 (0)
Missing	6 (25.0)	2 (10.4)	12 (42.9)	8 (34.9)
**Perceived economy, at Q2**				
Very good	2 (8.3)	3 (15.8)	1 (3.6)	1 (4.3)
Good	8 (33.3)	3 (15.8)	15 (53.6)	6 (26.2)
Sufficient	11 (45.9)	4 (21.1)	6 (21.4)	4 (17.4)
Strained	2 (8.3)	0 (0)	0 (0)	1 (4.3)
Missing	1 (4.2)	9 (47.3)	6 (21.4)	11 (47.8)
**Employment, at Q2**				
On parental leave	22 (91.7)	0 (0)	20 (71.4)	2 (8.7)
Other	1 (4.2)	10 (52.6)	2 (7.1)	10 (43.5)
Missing	1 (4.2)	9 (47.4)	6 (21.4)	11 (47.8)

*Questionnaires: Q1 First week after birth; Q2 Six months after birth*.

*Values: n = Number of participants; M, Mean; SD, Standard deviation*.

### Ethical Considerations

The Regional Ethical Review Board in Gothenburg, Sweden, approved this study (Dnr: 275–15). All participants were provided with information about the study and their right to withdraw their participation at any time. The participants gave written consent. Before data analysis, each questionnaire was stripped of identifiers and coded. Also, the results are reported on a group level, which makes it impossible for the reader of this article to identify the answers of a specific participant.

## Results

Eligible for analysis were first-time mothers and partners who responded to questionnaires at Q1 (IG first-time mothers *n* = 24, partners *n* = 19; CG first-time mothers *n* = 28, partners *n* = 23) and Q2 (IG first-time mothers *n* = 23, partners *n* = 10; CG first-time mothers *n* = 22, partners *n* = 12), as presented in Figure 1.

The socio-demographic characteristics of the participants are described in Table 2. A significant difference was observed between first-time mothers' age in IG and CG. No further significant differences were observed between the two study groups (results are not presented in tables). The index and dimensions were calculated for the different measurements included within this study; the results are presented in Table 3.

**Table 3:** Overview of index, dimensions and outcome measures at different times throughout the study.

	**1 week after childbirth (Q1)**						**6 months after childbirth (Q2)**					
	**IG**			**CG**			**IG**			**CG**		
	**All participants** **(*n* = 43)**	**First-time mothers** **(*n* = 24)**	**Partners** **(*n* = 19)**	**All participants** **(*n* = 51)**	**First-time mothers** **(*n* = 28)**	**Partners** **(*n* = 23)**	**All participants** **(*n* = 43)**	**First-time mothers** **(*n* = 24)**	**Partners** **(*n* = 19)**	**All participants** **(*n* = 51)**	**First-time mothers** **(*n* = 28)**	**Partners** **(*n* = 23)**
	**Mean (SD) α**	**Mean (SD)**	**Mean (SD)**	**Mean (SD) α**	**Mean (SD)**	**Mean (SD)**	**Mean (SD) α**	**Mean (SD)**	**Mean (SD)**	**Mean (SD) α**	**Mean (SD)**	**Mean (SD)**
Positive birth experience	3.3 (1.0)	3.3 (1.0)	3.2 (1.0)	3.5 (0.8)	3.3 (0.8)	3.6 (0.8)	3.5 (0.9)	3.5 (1.0)	3.7 (0.5)	3.3 (0.9)	3.2 (1.0)	3.5 (0.8)
Meaningfulness of a perceived positive birth experience	3.3 (0.9)	3.5 (0.8)	3.0 (1.0)	3.4 (0.7)	3.7 (0.8)	3.1 (0.8)	3.3 (0.8)	3.3 (0.8)	3.4 (0.7)	3.2 (1.0)	3.5 (0.7)	2.7 (1.2)
Perception of a normal birth	3.0 (1.2)	3.1 (1.3)	3.0 (1.1)	3.2 (1.0)	3.3 (0.9)	3.2 (1.1)	3.2 (1.1)	3.1 (1.1)	3.3 (0.7)	3.2 (1.1)	3.2 (1.1)	3.3 (1.1)
Meaningfulness of a perceived normal birth	3.2 (1.0)	3.4 (0.8)	3.0 (1.2)	3.5 (0.7)	3.6 (0.5)	3.3 (0.8)	1.9 (0.9)	1.9 (0.9)	1.8 (0.8)	1.8 (1.0)	1.6 (1.0)	2.1 (1.1)
Feelings of being safe during birth	3.5 (1.1)	3.2 (0.9)	3.8 (1.2)	3.4 (0.7)	3.5 (0.5)	3.3 (0.9)	5.2 (1.0)	5.3 (1.0)	5.0 (0.9)	5.3 (1.0)	5.2 (1.2)	5.4 (0.5)
Feelings of having control during birth	3.6 (1.7)	3.6 (1.6)	3.7 (1.9)	3.1 (1.7)	2.5 (1.4)	3.6 (1.9)	4.3 (1.3)	4.5 (1.4)	4.0 (0.9)	3.9 (1.4)	4.2 (1.3)	3.5 (1.4)
Skin-to-skin contact after birth^a^	65.1% (*n =* 28)	62.5% (*n =* 15)	68.4% (*n =* 13)	47.1% (*n =* 24)	42.9% (*n =* 12)	52.2% (*n =* 12)						
MoPPS index labor ward	45.4 (8.7) 0.88	45.5 (8.2)	45.2 (9.6)	48.8 (5.1) 0.76	47.9 (5.2)	49.8 (5.1)						
MoPPS index postnatal ward	47.4 (8.2) 0.54	47.7 (7.4)	46.8 (9.5)	52.0 (5.6) 0.51	51.2 (6.2)	53.5 (3.9) 8						
QDR36 index	25.3 (2.1) 0.89	25.4 (2.3)	25.2 (1.9)	25.1 (2.9) 0.94	25.0 (3.3)	25.1 (2.7)	24.3 (2.6) 0.92	24.3 (2.9)	24.5 (1.6)	24.1 (2.5) 0.91	24.6 (2.5)	23.2 (2.1)
**Dimensions**												
Consensus	5.4 (0.4)	5.4 (0.3)	5.3 (0.4)	5.2 (0.5)	5.3 (0.6)	5.2 (0.5)	5.2 (0.5)	5.2 (0.5)	5.4 (0.4)	5.2 (0.4)	5.3 (0.4)	5.0 (0.4)
Cohesion	5.3 (0.6)	5.3 (0.6)	5.3 (0.6)	5.3 (0.7)	5.4 (0.8)	5.2 (0.7)	4.9 (0.8)	4.8 (0.8)	5.1 (0.8)	5.0 (0.7)	5.2 (0.7)	4.8 (0.7)
Satisfaction	5.3 (0.4)	5.3 (0.4)	5.3 (0.5)	5.3 (0.5)	5.3 (0.5)	5.3 (0.4)	5.1 (0.4)	5.1 (0.5)	5.1 (0.3)	5.1 (0.4)	5.1 (0.5)	5.1 (0.3)
Sensuality	5.2 (0.7)	5.3 (0.8)	5.1 (0.7)	5.2 (0.8)	5.3 (0.8)	5.1 (0.8)	5.1 (0.9)	5.1 (0.9)	5.2 (0.7)	5.1 (0.8)	5.1 (0.9)	4.9 (0.7)
Sexuality	4.1 (0.7)	4.2 (0.5)	4.1 (0.8)	4.0 (1.0)	4.0 (1.0)	4.0 (1.0)	4.1 (0.8)	4.1 (0.8)	4.0 (0.6)	3.6 (0.9)	3.7 (0.9)	3.4 (0.8)
MSPSS index	79.7 (5.8) 0.92	80.7 (5.1)	78.6 (6.4)	80.0 (5.5) 0.89	80.6 (5.8)	79.4 (5.3)	76.6 (7.6) 0.90	76.7 (8.0)	76.1 (6.9)	73.6 (11.3) 0.94	77.6 (6.8)	66.8 (14.2)
**Dimensions**												
Family	26.4 (2.4)	27.0 (1.8)	25.8 (2.8)	27.0 (1.7)	27.1 (1.9)	26.9 (1.6)	25.4 (4.0)	25.7 (3.9)	24.5 (4.1)	24.5 (3.9)	25.8 (2.4)	22.1 (4.9)
Friends	25.6 (3.1)	25.9 (3.3)	25.2 (3.1)	25.4 (3.5)	25.7 (3.7)	25.0 (3.3)	24.1 (3.5)	24.0 (3.5)	24.5 (3.6)	23.4 (5.5)	24.5 (5.2)	21.6 (5.8)
Significant others	27.7 (1.0)	27.8 (0.6)	27.6 (1.3)	27.7 (0.8)	27.8 (0.8)	27.5 (0.9)	27.0 (2.1)	27.0 (2.3)	27.0 (1.6)	25.7 (3.8)	27.1 (1.5)	23.2 (5.3)
SOC-13 index	73.8 (10.0) 0.87	74.7 (9.2)	72.9 (10.9)	70.5 (8.1) 0.78	72.9 (7.0)	68.0 (8.7)	70.9 (9.4) 0.81	69.6 (9.5)	73.9 (8.9)	70.8 (9.6) 0.83	72.6 (9.3)	67.8 (9.8)
**Dimensions**												
Comprehensibility	27.5 (4.3)	27.4 (3.9)	27.7 (4.9)	26.2 (3.8)	27.3 (3.1)	25.1 (4.1)	26.4 (4.3)	25.8 (4.3)	27.8 (4.2)	25.9 (5.1)	26.4 (5.4)	25.0 (4.8)
Manageability	22.7 (3.0)	22.7 (2.8)	22.8 (3.2)	21.4 (2.7)	22.1 (2.6)	20.7 (2.8)	21.7 (3.3)	21.1 (3.4)	23.0 (3.0)	21.5 (3.3)	21.9 (3.2)	21.0 (3.5)
Meaningfulness	23.5 (3.7)	24.6 (3.4)	22.4 (3.7)	22.9 (2.8)	23.4 (2.3)	22.3 (3.2)	23.0 (3.1)	23.0 (3.1)	23.1 (3.1)	23.4 (3.4)	24.3 (2.8)	22.0 (4.0)
Parent's perceived relation to the child index		41.9 (3.9) 0.65	36.1 (3.5) 0.57		43.4 (3.8) 0.50	35.7 (25) 0.60		44.9 (2.4) 0.30	36.4 (3.5) 0.67		44.6 (3.1) 0.39	35.3 (3.8) 0.60
Parent's perceived feelings for the child index	44.9 (4.6) 0.87	44.8 (4.0)	45.1 (5.2)	46.4 (2.9) 0.56	46.5 (3.3)	46.3 (2.5)	47.3 (2.7) 0.87	47.6 (2.5)	46.5 (3.0)	47.4 (2.7) 0.78	48.0 (2.0)	46.4 (3.4)

*Results from Cronbach's alpha test included for index*.

Response mean values:

*Positive birth experience: theoretical range 1–4*.

*Meaningfulness of a perceived positive birth experience: theoretical range 1–4*.

*Perception of a normal birth: theoretical range 1–4*.

*Meaningfulness of a perceived normal birth: theoretical range 1–4*.

*Feelings of safety during birth: theoretical range 1–6*.

*Feelings of having control during birth: theoretical range 1–6*.

a *Skin-to-skin contact after birth: a variable with two categories: (1) skin-to-skin contact during the first 2 h after birth and (2) no skin-to-skin contact during the first 2 h after birth*.

*MoPPS index: theoretical range 8–56*.

*QDR36 index: theoretical range 5–30, dimensions: range 1–6*.

*MSPSS index: theoretical range 12–84, dimensions: range 4–28*.

*SOC-13 index: theoretical range 13–91, dimensions: range Comprehensibility (5 items) range 5–35; Manageability (4 items) range 4–28; Meaningfulness (4 items) range 4–28*.

*Parent's perceived relation to the child index: For first-time mothers (7 items, ‘Enjoy to breastfeed’ included in index): theoretical range 7–49: For partners (6 items, “Enjoy to breastfeed” excluded from index): theoretical range 6–42*.

*Parent's perceived feelings for the child index: theoretical range 7–49*.

*Values: n, number of participants; M, mean; SD, standard deviation; α, Cronbach's alpha*.

### Effects of the Intervention for First-Time Mothers and Partners

In relation to the primary outcome, the results showed a tendency to a *positive* effect from the intervention on the first-time mothers' feelings of having control during birth at Q1 (U = 90.0, *p* = 0.096) with a large clinical effect size (Table 4).

**Table 4:** Results from the Mann-Whitney test for comparison between IG and CG among first-time mothers and partners first week (Q1) and 6 months after birth (Q2).

	**1 week after childbirth (Q1)**			**6 months after childbirth (Q2)**			**Change between Q1 and Q2**		
	**Total** **(IG/CG)** **U(*p-*value) η^2^**	**First-time mothers** **(IG/CG)** **U (*p-*value) η^2^**	**Partners** **(IG/CG)** **U (*p-*value) η^2^**	**Total** **(IG/CG)** **U(*p-*value) η^2^**	**First-time mothers** **(IG/CG)** **U (*p-*value) η^2^**	**Partners** **(IG/CG)** **U (*p-*value) η^2^**	**Total** **(IG/CG)** **U (*p-*value) η^2^**	**First-time mothers** **(IG/CG)** **U (*p-*value) η^2^**	**Partners** **(IG/CG)** **U (*p-*value) η^2^**
Positive birth experience	500.0 (0.536)	132.5 (0.658)	97.5 (0.190)	450.5 (0.159)	188.0 (0.149)	55.0 (0.688)			
Meaningfulness of a perceived positive birth experience	519.5 (0.745)	133.5 (0.660)	125.5 (0.936)	538.0 (0.928)	197.0 (0.245)	38.0 (0.122)			
Perception of a normal birth	508.5 (0.635)	140.0 (0.880)	115.0 (0.611)	513.5 (0.654)	227.5 (0.714)	57.5 (0.848)			
Meaningfulness of a perceived normal birth	507.5 (0.618)	133.0 (0.659)	119.5 (0.745)	485.5 (0.416)	185.0 (0.149)	53.0 (0.623)			
Feelings of being safe during birth	500.5 (0.889)	105.0 (0.229)	89.0 (0.189)	535.5 (0.899)	226.5 (0.695)	46.0 (0.299)			
Feelings of having control during birth	433.0 (0.218)	90.0 (0.096#) >0.14	122.0 (0.833)	446.5 (0.197)	200.0 (0.315)	47.0 (0.376)			
Skin-to-skin contact after birth	0.00 (0.999)	130.5 (0.808)	123.0 (0.812)						
MoPPS index labor ward	365.5 (0.162)	116.0 (0.491)	72.0 (0.238)						
MoPPS index postnatal ward	224.5 (0.011^*^) >0.14	96.0 (0.094#) >0.14	27.0 (0.062#) >0.14						
QDR36index	492.5 (0.840)	133.0 (0.942)	115.5 (0.889)	417.0 (0.489)	215.0 (0.900)	26.0 (0.137)	168.0 (0.415)	86.0 (0.921)	10.0 (0.116)
**Dimensions**									
Consensus	462.0 (0.299)	131.0 (0.652)	104.0 (0.373)	474.0 (0.477)	221.0 (0.628)	26.5 (0.050^*^) >0.06			
Cohesion	502.0 (0.759)	123.0 (0.458)	114.0 (0.841)	508.0 (0.637)	189.0 (0.215)	43.0 (0.256)			
Satisfaction	538.0 (0.954)	135.0 (0.755)	124.5 (909)	464.0 (0.399)	198.0 (0.435)	56.5 (0.816)			
Sensuality	521.5 (0.785)	136.5 (0.793)	125.5 (0.939)	456.0 (0.449)	230.0 (0.980)	37.0 (0.201)			
Sexuality	504.0 (0.781)	126.5 (0.757)	126.0 (0.955)	366.5 (0.033^*^) >0.14	180.5 (0.150)	31.0 (0.100)			
MSPSS index	504.0 (0.774)	143.5 *(0.9*85)	110.5 (0.731)	452.0 (0.419)	220.5 (0.816)	31.0 (0.100)	169.0 (0.353)	83.0 (0.920)	12.5 (0.124)
**Dimensions**									
Family	454.5 (0.294)	135.5 (0.716)	92.5 (0.262)	425.5 (0.115)	196.5 (0.262)	43.0 (0.257)			
Friends	523.0 (0.978)	141.0 (0.911)	114.5 (0.855)	526.5 (0.984)	181.0 (0.228)	39.5 (0.170)			
Significant others	500.0 (0.584)	138.0 (0.674)	101.5 (0.314)	434.5 (0.151)	227.0 (0.672)	30.5 (0.070#) >0.06			
SOC-13 index	405.0 (0.077#) >0.14	116.5 (0.342)	89.5 (0.151)	525.5 (0.974)	185.5 (0.268)	38.0 (0.146)	189.0 (0.339)	57.5 (0.132)	26.0 (0.527)
**Dimensions**									
Comprehensibility	437.0 (0.174)	139.5 (0.976)	85.5 (0.112)	494.0 (0.655)	211.5 (0.635)	39.5 (0.175)			
Manageability	389.5 (0.048^*^) >0.14	123.0 (0.466)	71.5 (0.033^*^) >0.14	525.5 (0.806)	215.5 (0.539)	41.0 (0.207)			
Meaningfulness	435.0 (0.165)	100.5 (0.131)	117.0 (0.689)	485.5 (0.446)	178.5 (0.136)	52.0 (0.594)			
^a^Parent's perceived relation to the child index		84.5 (0.274)			197.0 (0.935)	51.0 (0.551)			
Parent's perceived feelings for the child index		98.0 (0.174)			229.0 (0.953)	58.5 (0.917)			

Measurements:

a *Parent's perceived relation to the child index: For first-time mothers, the question regarding breastfeeding was included, score index 7–49; For partners, the question regarding breastfeeding was excluded, score index 6–42*.

p-values:

* *p < 0.05 two tailed*.

Tendencies:

# *p ≤ 0.1*. *η^2^ interpretation sensu Cohen (48): Calculated for significant results from the Mann-Whitney test. >0.01 small effect, >0.06 medium effect; >0.14 large effect*.

In relation to the secondary outcomes, the results revealed that parents within the CG reported significantly *stronger* perceived support than parents within the IG (U = 224.5, *p* = 0.011) with a large effect size. Furthermore, the results revealed that the intervention had a significant *positive* effect on parents' (IG) perceived dyadic sexuality (dimension within QDR36) at Q2 (U = 366.5, *p* = 0.033) with a large effect size. Parents within the IG reported a *stronger* manageability (dimension within SOC-13) at Q1 (U = 389.5, *p* = 0.048) with a large effect size. Also, there was a tendency for a *positive* effect of the intervention on parents' (IG) SOC-13 index at Q1 (U = 405.0, *p* = 0.077), with a large effect size (Table 4).

When comparing differences between first-time mothers and partners separately, the results showed a *positive* effect in the IG intervention on partners' manageability (dimension within SOC-13) at Q1 (U = 71.5, *p* = 0.033) with a large effect size and consensus (dimension within QDR36) at Q2 (U = 26.5, *p* = 0.050) with a medium effect size (Table 4). For the inspiration lecture, no effectiveness was found with regard to obstetric and neonatal outcomes (Table 5), breastfeeding (Table 6), skin-to-skin contact after birth (Table 4), or the parents' perceived relation and feelings for the child (Table 4).

**Table 5:** Data for birth outcome collected through the first-time mothers' hospital medical records and results from the Mann-Whitney test.

	**First-time mothers** **IG** **(*n =* 24)** **Mean (SD)**	**First-time mothers** **CG** **(*n =* 28)** **Mean (SD)**	**First-time mothers** **(IG/CG)** **U (*p-*value)**
Length of birth (h)^a^	12.3 (6.3)	15.4 (10.6)	112.5 (0.559)
Dilation of cervix when arriving to labor ward (cm)	5.9 (3.0)	5.0 (3.0)	69.0 (0.436)
Vaginal/Instrumental birth^b^, *n (%)*	14 (78.0)/4 (22.0)	12 (75.0)/4 (25.0)	140.0 (0.851)
Time between arrival at labor ward and birth (h)	10.3 (14.5)	11.7 (11.0)	126.0 (0.534)
Gestational week at birth	39.8 (1.2)	39.9 (1.9)	121.5 (0.424)
Apgar Score at 5 min	9.5 (1.2)	9.6 (0.8)	129.0 (0.486)

a *Length of birth: time from start of contractions (first stage of labor/latent phase) to birth, in hours*.

b *Vaginal/Instrumental birth: a variable with two categories: (1) vaginal spontaneous birth and (2) vacuum extraction, forceps or section*.

**Table 6:** Breastfeeding descriptives and results from Mann-Whitney.

	**1 week after childbirth (Q1)**			**6 months after childbirth (Q2)**		
	**First-time mothers** **IG** **(*n =* 24)** ***n* (%)**	**First-time mothers** **CG** **(*n =* 28)** ***n* (%)**	***p-*value**	**First-time mothers** **IG** **(*n =* 24)** ***n* (%)**	**First-time mothers** **CG** **(*n =* 28)** ***n* (%)**	***p-*value**
**Breastfeeding during first 2 h after birth^a^**						
Yes	12 (50.0)	9 (32.1)	109.5 (0.418)			
Tried	4 (16.7)	3 (10.7)				
No	1 (4.2)	4 (10.7)				
**Any breastfeeding^b^**						
Yes	16 (66.7)	14 (50.0)	126.5 (0.928)	14 (58.3)	13 (46.4)	239.0 (0.944)
No	1 (4.2)	1 (3.6)		9 (37.5)	8 (28.6)	
**Breastfeeding**						
Exclusive	12 (50.0)	10 (35.7)		9 (37.5)	6 (21.4)	
Partly	3 (12.5)	3 (10.7)		6 (25.0)	7 (25.0)	
Enjoy to breastfeed, mean (SD) *n*	5.8 (1.4) 16	6.0 (1.3) 12	98.5 (0.593)	2.4 (1.8) 20	2.5 (1.5) 20	188.0 (0.735)

a *Breastfeeding during the first 2 h after birth: (1) No, I did not breastfeed the first 2 h after birth; (1) I tried to breastfeed but did not succeed to; (3) Yes, I breastfed the first 2 h after birth*.

b *Breastfeeding: Any breastfeeding, both exclusively and partially breastfeeding included*.

*Enjoy to breastfeed: theoretical range 1–7*.

### Change Over Time in the Birth Experience, Perceived Quality of Parental Couple Relationship, Social Support, and SOC

The change in scores between the first week (Q1) and 6 months (Q2) after birth was calculated and analyzed. In relation to the primary outcome and the longitudinal effect of the intervention, the following significant results were revealed: (1) both parents within the IG and CG reported *lower* scores related to the meaningfulness of a perceived normal birth at Q2 in comparison with Q1 and (2) the parents' reported feelings of being safe during birth was *higher* at Q2 in comparison with Q1. In relation to the secondary outcomes, a significant difference was found in that the QDR36 index *decreased* between Q1 and Q2 among all parents within the CG (*p* = 0.019), as well as among partners separately within the CG (*p* = 0.028). The MSPSS index *decreased* between Q1 and Q2 among all parents within the IG (*p* = 0.001) and CG (*p* = 0.024), as well as among first-time mothers separately within the IG (*p* = 0.013). For change in the SOC-13 index between Q1 and Q2, there were no significant results. The results are presented in Table 7.

**Table 7:** Change over time in index and dimensions throughout the study between participants in IG and CG.

	**Change between Q1 and Q2** **IG**			**Change between Q1 and Q2** **CG**		
	**All participants**	**First-time mothers**	**Partners**	**All participants**	**First-time mothers**	**Partners**
	***p*-value**	***p*-value**	***p*-value**	***p*-value**	***p*-value**	***p*-value**
Positive birth experience	ns	ns	ns	ns	ns	ns
Meaningfulness of a perceived positive birth experience	ns	ns	ns	ns	ns	ns
Perception of a normal birth	ns	ns	ns	ns	ns	ns
Meaningfulness of a perceived normal birth	0.001^***^	0.005^**^	ns	0.002^**^	0.004^**^	0.136
Feelings of being safe during birth	0.000^***^	0.000^***^	0.067	0.000^***^	0.003^**^	0.008^**^
Feelings of having control during birth	0.101	0.195	ns	ns	0.023	ns
QDR36 index	ns	ns	ns	0.019^*^	ns	0.028^*^
MSPSS index	0.001^***^	0.013^*^	ns	0.024^*^	0.085	ns
SOC-13 index	ns	ns	ns	ns	ns	ns

*Results from non-parametric test (Wilcoxon Signed Rank test)*.

p-values:

* *p ≤ 0.05*,

** *p ≤ 0.01*,

*** *p ≤ 0.001, ns, non-significant Friedman's test*.

*Tendencies: #p ≤ 0.1*.

### Internal Consistency of the Measurements

Cronbach's alpha was calculated to evaluate the internal consistency for different measurements included in the study. The results showed high values for several of the included measures (MoPPS index labor ward; QDR36 index; MSPSS index; SOC-13 index; and parents' perceived feelings for the child index) and lower values for the MoPPS index postnatal ward, the parents' perceived relation to and feelings for the child index (Table 3).

## Discussion

In this trial of the inspirational lecture provided as a professional support for expectant parents in combination with ordinary antenatal parental classes, we observed a tendency for an effect on first-time mothers' feelings of having control during birth. However, the effects of the intervention seemed to be more prominent for partners. The results suggest positive effects on the IG parents' couple relationship, such as sexuality for both mothers and partners, as well as the couple's consensus for partners. The dimension of consensus within QDR36 relates to the couple's ability to respond to common stimulation in the exchange of ideas, laughter, or discussions, and so on (3). Therefore, these findings are in line with earlier studies that the inspirational lecture can facilitate partners' engagement in preparation for childbirth and parenthood (19, 20), and first-time mothers perceive higher quality within the couple's relationship when partners show positive feelings for parenthood (9). These findings contribute valuable knowledge since the transition to parenthood is a period of vulnerability (49, 50), and parents need both professional and social support (19, 20). It is known that the quality of a parental couple's relationship generally decreases after birth (3, 4, 9, 11, 12), which could be due to the challenge of the transition to parenthood (3, 10, 24). If the inspirational lecture in combination with ordinary antenatal parental classes could have effects that strengthen the relationship of the parental couple, it could be a valuable intervention to introduce on a wide scale in society. However, the longitudinal effects of the combination between the inspirational lecture and ordinary antenatal parental classes on parents' strengthened couple relationship beyond 6 months after birth need further exploration.

Intervention in this pilot study had a positive effect on the SOC dimension of IG parents' and IG partners' manageability. This is a valuable result since a high SOC previously has been described as important for how parents perceive and cope with the challenges that come with childbirth and parenthood (15, 51). The fact that the combination of the inspirational lecture and ordinary antenatal parental classes led to the parents' higher manageability highlights the importance of a combination of different types of professional support for expectant parents. This is because manageability deals with the parents' overall sense that life is filled with meaning and purpose (16, 52), which is valuable during the transition to parenthood. Previously, parents' SOC has been described as increasing after birth (9, 53–55). However, the results of the present study showed no significant change in the parents' SOC between the first week and 6 months after birth. This result could be due to the small number of parents included in the present study. Therefore, it is suggested that both changes in SOC during the transition to parenthood and effects of the intervention on SOC beyond 6 months after birth be further explored.

The result that both IG and CG parents reported a decrease in perceived social support is in line with the results from a previous longitudinal cohort study, which also reveals that social support is associated with a higher quality of parental couples' relationships 6 months after birth (9). This stresses the value of group dialogue during parents-to-be meetings in antenatal parental classes. During such classes, expectant parents can relate the challenge of parental transition to others' experiences, which is described positively (19, 20). The intervention included in this study, the inspirational lecture, is a large-group lecture, and parents are not encouraged to interact with each other during the lecture. During the ordinary parental antenatal classes included in this study, the parents were, on the other hand, encouraged to discuss different issues regarding childbirth and parenthood in smaller groups of parents. According to other studies, first-time fathers experience exclusion by midwives who should offer both parents the opportunity to pose questions. It is also important to expectant fathers that time for discussion is included in antenatal parental classes (22), and first-time fathers require child health nurses' support to adapt to their role of fatherhood (38). As mentioned, the inspirational lecture does not promote interaction between attending couples, which could be an area for development of the concept. Further, the results revealed that parents in the CG perceived stronger professional support from professionals in the postnatal ward than parents in the IG. This is not in line with earlier research that shows that extended professional support during pregnancy will improve perceived professional support after birth (35). One explanation for this result could be that the parents within the IG had higher manageability and therefore were less in need of professional support in the postnatal ward. However, childbirth is complex and more research is needed to fully understand the effects of the inspirational lecture as a complement to ordinary antenatal parental classes.

The intervention tended to produce positive effects on IG mothers' feelings of having control during birth. These results point to an important finding since a woman's birth experiences will influence her throughout her life (56). A positive birth experience has a good impact for both the woman and the baby's well-being but also for the couple's relationship (57). One reason for IG mothers' feelings of having control during birth could be that the inspirational lecture also had positive effects on partners' SOC and parental couple's relationships. This could lead to a partner being more able to support integrative power during birth. Integrative power means to support the woman's ability to surrender herself to the power of physiological birth (58) and thereby have a feeling of control yet surrendering to the process (56). A trusting relationship with her partner during birth is important for a woman (59, 60) and contributes to each woman feeling safe (34), plus her relationship with her partner may be strengthened (61). Feeling safe during birth has positive effects on mothers' breastfeeding (62). The results of this study showed no significant results for the analysis concerning breastfeeding. This could, however, be due to the relatively small data available. In addition, birth is complex, and more research is needed to fully understand how different processes interact. In this pilot study, we evaluated the effects of expectant parents receiving a combination of the inspirational lecture and ordinary antenatal parental classes compared with expectant parents receiving only ordinary antenatal parental classes. To gain deeper knowledge about the inspirational lecture as a professional support for parents-to-be, further research is needed on the midwives' experiences from providing the lecture. The present study might be an example of how a work-integrated learning (WIL) perspective can be valuable to further explore the pedagogical approach used by the midwives providing the lecture. Future research, including a WIL perspective with specialization in healthcare pedagogics, may contribute to improved high-quality care that meets both current and future needs (63) among parents-to-be.

Randomized controlled trials are viewed as the gold standard in evaluating interventions. In this pilot study, the sampling plan was predisposed by a time aspect, and consecutive sampling was performed. Consecutive sampling has previously been described to reduce the risk of bias (32). Conducting intervention studies within clinical settings is, however, a challenge. In this study, the recruitment of participants varied from including almost all eligible parents to including around half or less of eligible parents. This variation was, among other things, explained by the high workload of the midwives at one of the antenatal clinics. Subsequently, we did not reach the targeted number of participants. Therefore, when considering the results it is important to bear in mind that this is a pilot study, and one explanation for why there is no convincing evidence of the effects of the intervention might be the small sample size. Also, we did not estimate any power analysis for this study, which could be seen as a study limitation, and the relatively short sampling period could be a potential risk for bias concerning seasonal or other time-related fluctuations (64). However, the participants within the IG and KG were relatively homogeneous, and a small sample may, therefore, be adequate (32). Also, based on previous qualitative research on parents' perceptions of the inspirational lecture (19, 20), there are reasons to expect that the independent and dependent variables will be strongly related. Despite the small sample size, this study has some interesting findings that add new knowledge to the field, implying that the inspirational lecture in combination with ordinary antenatal parental classes could be a valuable care intervention in parental preparatory professional support. Nevertheless, the results should be interpreted with caution considering the number of parents who were not invited or who declined participation. Also, when conducting intervention studies, including interventions that are not possible to blind for the participants, there might be a risk of potential desirability bias. In this study, it could be the case that the parents who “won” the ticket to extra professional support (the inspirational lecture), where thankful and wanted to give something back to the midwives providing the lecture. However, we think that such potential study bias should have been more apparent in the results.

Further, study limitations were the lack of analysis of non-responders and baseline characteristics before the intervention. In contrast, the dropout rate was relatively low and the follow-up design could be considered as a study strength. Before conducting this study, two pilot studies were performed to test the questions and measurement clarity, as well as to test the technical issues relating to the web-based questionnaire, which could be considered as a study strength. One advantage of using the web-based questionnaire for Q2 was that participants' answers could be directly transferred to SPSS. For Q1, participants' answers were manually transferred to SPSS. To be able to reduce the risk of errors from the manual transfer, data in SPSS was carefully controlled with the participants' answers in Q1. The lesson learned from this was that web-based questionnaires should be preferable to use in future studies.

When designing this study, we used a follow-up design including repeated questionnaires, several measurements, and analyses. The reason for this was the fact that childbirth and becoming parents are life-changing experiences for the parents, and the inspirational lecture has previously been shown to have an impact on parents' feelings of being prepared for childbirth and parenthood (19, 20). When designing this pilot study, our intent was to be able to analyze the possible effects that the intervention would have on different aspects valuable for the parents, such as their birth experiences, sense of coherence, perceptions of social and professional support, and parental couple relationship. Including several measurements made it possible to, somehow, embrace the complex processes of childbirth and parental transition. However, childbirth and becoming parents are complex processes, and more research is needed to fully comprehend all aspects. Future intervention studies, evaluating the effects of professional support, are needed, and the design of such studies should be carefully considered. For better opportunities to reach statistical power and higher generalizability, a multicenter design could be preferable, for example. For future studies, the total scores obtained from the psychometric instruments in this study could be used as comparison scores for the target group. Furthermore, when evaluating the internal consistency of the included different measurements, the results showed high values for several of the measures, and lower values were shown for the MoPPS and MIRF scale, which indicates the need for improvement of the items in future studies. Within this study, we have analyzed both the index and dimensions for different measurements, as well as a few items from different measures. Therefore, it is important to interpret our results with care.

## Conclusions and Clinical Implications

The results from this pilot intervention study revealed that a combination of professional parental preparatory support between the inspirational lecture and ordinary antenatal parental classes showed a tendency to be gainful on parents' feelings of having control during birth, parental couples' relationships, and SOC, while some results were more prominent for partners. However, this professional support intervention did not strengthen social support, which all professional support actions should aim to do. Despite the small sample, this study revealed that the concept of the inspirational lecture as large-group parental preparatory professional support seems to be a valuable care intervention in combination with ordinary antenatal parental classes. More research is needed since childbirth and transition to parenthood are complex processes that need to be comprehended, and care interventions could preferably be evaluated using a multicenter design in the future.

## Data Availability Statement

The datasets generated for this study will not be made publicly available because the authors don't have permission to share the data.

## Ethics Statement

The studies involving human participants were reviewed and approved by the Regional Ethical Review Board in Gothenburg, Sweden (Dnr: 275-15). The patients/participants provided their written informed consent to participate in this study. Written informed consent was obtained from the individual(s) for the publication of any potentially identifiable images or data included in this article.

## Author Contributions

ST contributed to conceptualization, data curation, formal analysis, funding acquisition, investigation, methodology, project administration, resources, software, validation, visualization, and writing of the article, which is an original draft. AE-B contributed to conceptualization, formal analysis, funding acquisition, methodology, validation, visualization, and writing of the article, which is an original draft. CB contributed to conceptualization, formal analysis, funding acquisition, investigation, methodology, resources, software, validation, visualization, and writing of the article, which is an original draft. All authors contributed to the article and approved the submitted version.

## Conflict of Interest

The authors declare that the research was conducted in the absence of any commercial or financial relationships that could be construed as a potential conflict of interest. The reviewer MG declared a shared affiliation, though no other collaboration, with one of the authors AE-B to the handling Editor.

## Abbreviations

MoPPS scale: Mother Perceived Professionals Support Scale
QDR36: Perceived Quality of Parental Couple Relationship Scale
MSPSS scale: The Multidimensional Scale of Perceived Social Support
SOC-13: Sense of Coherence, 13-Item Scale
MIRF scale: Mother to Infant Relations and Feelings scale.
